# Preoperative Systemic Inflammatory Marker Profile in Surgically Treated Intradural Spinal Tumors: A Retrospective Cohort Study

**DOI:** 10.3390/medicina62050950

**Published:** 2026-05-13

**Authors:** Muhammet Kırkgeçit, Hasan Türkoğlu, Muharrem Furkan Yüzbaşı, Emrullah Cem Kesilmez, Fırat Yıldız, Yusuf Aslan, Şahin Kırmızıgöz, Kasım Zafer Yüksel

**Affiliations:** 1 Department of Neurosurgery, Megapoint Hospital, Kahramanmaraş 46100, Turkey; 2 Department of Neurosurgery, Gaziantep City Hospital, Gaziantep 27470, Turkey; has.1453@hotmail.com (H.T.); tbp.sahin@gmail.com (Ş.K.); 3 Department of Neurosurgery, Kahramanmaraş Sütçü İmam University, Kahramanmaraş 46050, Turkey; dr.furkanyuzbasi@gmail.com (M.F.Y.); cemkesilmez@gmail.com (E.C.K.); kzyuksel@hotmail.com (K.Z.Y.); 4 Department of Neurosurgery, Aksaray Training and Research Hospital, Aksaray 68100, Turkey; firat.yildiz@aksaray.edu.tr; 5 Department of Neurosurgery, Osmaniye Training and Research Hospital, Osmaniye 80010, Turkey; op.dr.yusufaslan@gmail.com

**Keywords:** spinal tumors, inflammation, NLR, PLR, SII, PIV, biomarkers

## Abstract

*Background and Objectives*: We aimed to determine whether preoperative systemic inflammatory markers derived from complete blood count differ between patients with intradural spinal tumors and healthy controls, and whether any such difference varies by pathological subtype or motor deficit status. *Materials and Methods*: Sixty-four patients who underwent surgery for histopathologically confirmed intradural spinal tumors between 2015 and 2023 were enrolled alongside 64 age- and sex-matched healthy controls. The neutrophil-to-lymphocyte ratio (NLR), platelet-to-lymphocyte ratio (PLR), lymphocyte-to-monocyte ratio (LMR), systemic immune-inflammation index (SII), pan-immune-inflammation value (PIV), and red cell distribution width (RDW) were calculated from preoperative complete blood count results. Comparisons were performed at the patient–control level and stratified by pathological diagnosis (meningioma, schwannoma, ependymoma, other) and motor deficit status. *Results*: NLR (median 2.47 vs. 2.06; *p* < 0.001), PLR (157.1 vs. 121.0; *p* < 0.001), SII (706.1 vs. 595.0; *p* = 0.003), and PIV (404.2 vs. 287.0; *p* < 0.001) were all significantly elevated in the tumor group, while LMR was significantly lower (3.66 vs. 4.34; *p* < 0.001). RDW did not differ between groups (*p* = 0.420). Stratification by pathological subgroup and motor deficit status revealed no significant differences in any marker. *Conclusion*: Intradural spinal tumors—including the predominantly benign cases that made up most of this cohort—are accompanied by a detectable preoperative shift in systemic inflammatory markers, one that appears independent of tumor histology and neurological presentation. These findings demonstrate a measurable systemic inflammatory response in patients with intradural spinal tumors. However, the absence of differences across pathological subtypes and motor deficit status suggests that these markers reflect a generalized host response rather than tumor-specific characteristics, and their role in clinical decision-making remains to be clarified.

## 1. Introduction

Tumor development and progression are tightly coupled to the inflammatory milieu surrounding malignant cells and to the host’s systemic immune response. Complete blood count (CBC)-derived inflammatory indices offer a window into this interaction at virtually no additional cost, which partly explains why they have accumulated such a broad evidence base in oncology over the past decade. The neutrophil-to-lymphocyte ratio (NLR) is the most widely studied of these parameters; across numerous meta-analyses, elevated preoperative NLR consistently associates with adverse oncological outcomes [1]. The platelet-to-lymphocyte ratio (PLR) and lymphocyte-to-monocyte ratio (LMR) have been examined as complementary markers, and low LMR in particular has been linked to shortened survival in several malignancies [2,3]. Composite indices extend this logic further by integrating multiple cell lineages into a single value. The systemic immune-inflammation index (SII), calculated as neutrophil × platelet/lymphocyte, was first validated in hepatocellular carcinoma; the pan-immune-inflammation value (PIV), which additionally incorporates the monocyte count, has been associated with poor survival in metastatic colorectal cancer and in patients with advanced non-small cell lung cancer (NSCLC) receiving immunotherapy [4,5,6]. Red cell distribution width (RDW), a measure of erythrocyte volume heterogeneity, rounds out the picture as an inflammation-sensitive parameter independently linked to unfavorable outcomes across multiple cancer types [7,8].

What the literature lacks is breadth. Most studies focus on a single marker or on a single tumor type, and cross-histology comparisons remain scarce; subgroup-level interpretation is correspondingly difficult [1,2]. Even for PIV, promising data from selected tumor types sit alongside conspicuous gaps—the head and neck cancer population, for instance, has been largely left aside [9]. Spinal tumors represent a more pronounced blind spot: no study to date has examined the preoperative profile of NLR, PLR, LMR, SII, PIV, and RDW together in this population, let alone explored whether these markers track with pathological diagnosis or clinical neurological status.

Our central hypothesis was that preoperative systemic inflammatory markers would be measurably different in patients with intradural spinal tumors compared with healthy individuals, yet would not discriminate reliably between pathological subgroups or between patients with and without motor deficit. We set out to test whether these easily calculated ratios are shifted relative to controls, whether any separation exists across the meningioma, schwannoma, ependymoma, and other diagnostic groups, and whether the presence of motor deficit carries a distinct inflammatory signature. Systemic inflammatory markers such as NLR, PLR, and LMR have been widely studied in oncology and have been associated with tumor aggressiveness, prognosis, and survival outcomes in various malignancies, including central nervous system tumors. However, their ability to distinguish between histological subtypes or to reflect neurological status remains unclear. This gap in the literature provided the rationale for our hypothesis.

## 2. Materials and Methods

### 2.1. Study Population and Sample

This was a retrospective study of consecutive patients who underwent surgery for spinal tumors at our institution between 2015 and 2023. Eligible patients were adults (≥18 years) with a histopathologically confirmed intradural spinal tumor, available excisional or biopsy material, and retrievable preoperative complete blood count (CBC) data. Patients with active infection, a known hematological disorder, current immunosuppressive therapy, or pregnancy were excluded. Sixty-four patients met these criteria and were enrolled. A control group of 64 healthy individuals—32 male and 32 female, mean age 47.0 ± 10.3 years—was selected by matching for age and sex; none had a known malignancy, inflammatory disease, or immunosuppressive drug use. Recorded clinical variables included age, sex, lesion level (cervical, thoracic, or lumbar), pathological diagnosis (meningioma, schwannoma, ependymoma, or other), motor deficit status, surgical complications, and recurrence.

### 2.2. Study Procedures

Preoperative CBC was obtained from venous blood samples drawn within 24 h before surgery. Six systemic inflammatory markers were derived from these values. The neutrophil-to-lymphocyte ratio (NLR) reflects the balance between pro-inflammatory and antitumoral immune activity in the tumor microenvironment and carries independent prognostic value in solid tumors [1,10]. The platelet-to-lymphocyte ratio (PLR) captures platelet-mediated inflammatory activity and the tendency toward immune suppression, with prognostic relevance in cancer established across multiple series [2]. The lymphocyte-to-monocyte ratio (LMR), for which low values associate with poor prognosis in various malignancies, serves as a broader index of systemic immune balance [3]. The systemic immune-inflammation index (SII) is calculated as neutrophil × platelet/lymphocyte; originally described in hepatocellular carcinoma, it has since entered widespread use in oncology [4]. The pan-immune-inflammation value (PIV)—neutrophil × platelet × monocyte/lymphocyte—integrates all four peripheral blood lineages into a single composite score and, in patients with metastatic colorectal cancer, outperformed the other inflammatory indices in prognostic accuracy [5]. Finally, red cell distribution width (RDW, %), a measure of erythrocyte volume heterogeneity that rises with inflammatory cytokine activity, has been identified as an independent prognostic marker in several cancer types [7,8]. Motor deficit was assessed by preoperative neurological examination; complications and recurrence were ascertained from postoperative clinical and radiological follow-up records. All pathological diagnoses were confirmed by an experienced neuropathologist.

### 2.3. Surgical Technique

All patients underwent surgery under general anesthesia following standard microsurgical neurosurgical protocols. The extent of resection—total or subtotal—was determined by lesion location and pathological characteristics, with the final intraoperative decision made by the operating neurosurgeon. Patients were subsequently classified into four groups based on pathological diagnosis: meningioma, schwannoma, ependymoma, and other.

### 2.4. Statistical Analysis

Data were analyzed using SPSS version 26.0 (IBM Corp., Armonk, NY, USA). Normality was assessed with the Shapiro–Wilk test. Age did not follow a normal distribution in the overall sample (Shapiro–Wilk W = 0.959, *p* = 0.032), and none of the inflammatory markers met the normality assumption. Age was normally distributed in both motor deficit subgroups (Shapiro–Wilk: with deficit *p* = 0.092, without deficit *p* = 0.097), so an independent-samples *t*-test was used for that specific comparison. Because individual-level control data were unavailable, patient–control comparisons for NLR, PLR, LMR, SII, and PIV were performed using one-sample Wilcoxon signed-rank tests against the control group medians; RDW was compared by one-sample *t*-test. Age was similarly compared using the one-sample Wilcoxon signed-rank test. Differences across the four pathological groups were examined with the Kruskal–Wallis test, followed by Dunn post-hoc testing with Bonferroni correction where significance was reached. Categorical variables were evaluated by chi-square test, with Fisher’s exact test applied when more than 25% of expected cell frequencies fell below five. Marker differences between motor deficit groups were assessed with the Mann–Whitney U test. Normally distributed continuous variables are reported as mean ± SD; non-normally distributed variables as median (IQR: 25th–75th percentile); categorical variables as *n* (%). A *p*-value below 0.05 was considered statistically significant throughout. To address the potential confounding effect of age on inflammatory markers, additional analyses were performed. The relationship between age and inflammatory markers was evaluated using correlation analysis within the patient cohort.

### 2.5. Ethical Considerations

The study was approved by the Medical Research Ethics Committee of Kahramanmaraş Sütçü İmam University (approval date: 15 December 2025; decision no: 08). Individual written informed consent was waived by the ethics committee given the retrospective design; all patient data were processed after de-identification. The study was conducted in accordance with the principles of the Declaration of Helsinki (2013 revision).

## 3. Results

### 3.1. Demographic and Clinical Characteristics

The cohort comprised 64 patients, evenly split by sex (32 male, 32 female), with a median age of 51.5 years (IQR: 38.0–68.0). The control group had a mean age of 47.0 ± 10.3 years; the patient group was significantly older (*p* = 0.017), while sex distribution was identical between groups (*p* = 1.000, chi-square) (Table 1).

Lumbar localization was the most common, accounting for half the cohort (*n* = 32, 50.0%), followed by thoracic (*n* = 24, 37.5%) and cervical (*n* = 8, 12.5%) levels. Meningioma was the predominant diagnosis (*n* = 27, 42.2%), with schwannoma (*n* = 12, 18.8%), other diagnoses (*n* = 17, 26.6%), and ependymoma (*n* = 8, 12.5%) making up the remainder. Motor deficit was present in 16 patients (25.0%). Urinary incontinence, a surgical complication, and tumor recurrence were each rare, occurring in 1 (1.6%), 1 (1.6%), and 4 (6.3%) patients, respectively (Table 1).

### 3.2. Comparison of Inflammatory Markers with Controls

**Figure 1:**
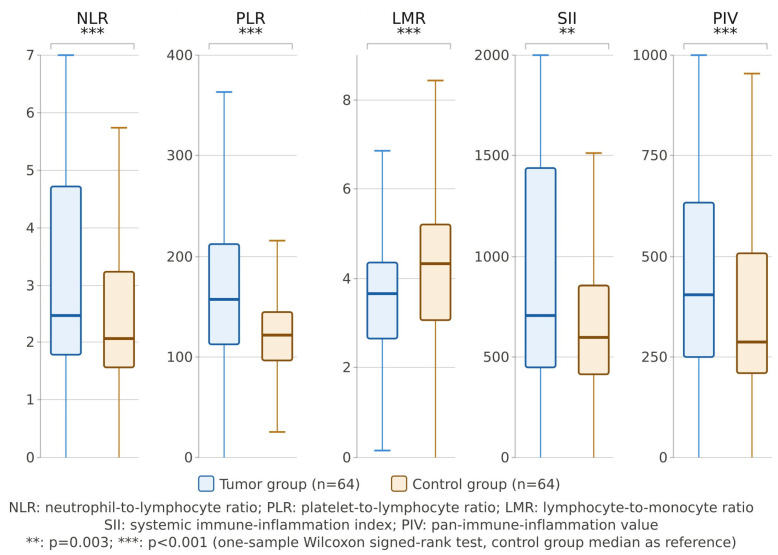
Systemic inflammatory markers in spinal tumor patients versus controls.

NLR (*p* < 0.001), PLR (*p* < 0.001), PIV (*p* < 0.001), and SII (*p* = 0.003) were all significantly elevated in the patient group relative to controls, while LMR was significantly lower (*p* < 0.001). RDW did not differ between groups (41.8 ± 4.3 vs. 42.3 ± 3.5; *p* = 0.420) (Table 2) (Figure 1).

### 3.3. Comparison Across Pathological Groups

**Figure 2:**
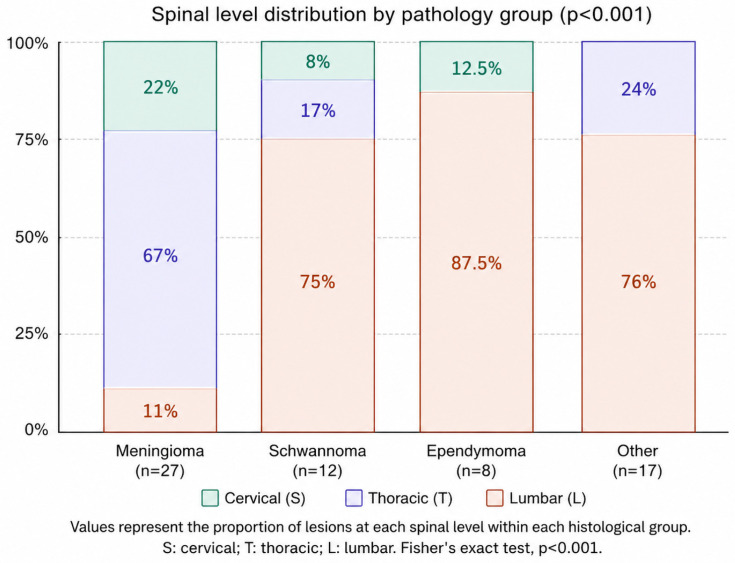
Spinal level distribution by pathology group (Fisher’s exact test, *p* < 0.001).

Age distribution differed significantly across the four pathological groups (*p* = 0.023). Ependymoma patients were the youngest, with a median age of 34.0 years, whereas the schwannoma group had the highest median age at 60.5 years. Lesion level distribution was markedly non-uniform (*p* < 0.001): meningiomas predominated at the thoracic level, while schwannomas and ependymomas were concentrated in the lumbar region (Figure 2). Sex distribution and motor deficit frequency did not differ across groups (*p* = 0.122 and *p* = 0.104, respectively).

Despite these demographic and anatomical differences, no marker separated the pathological subgroups. NLR, PLR, LMR, SII, PIV, and RDW were all statistically comparable across meningioma, schwannoma, ependymoma, and other diagnoses (all *p* > 0.05). The numerically highest NLR and SII values were observed in the meningioma and other groups, but these differences did not reach statistical significance (Table 3).

**Table 3:** Demographic, clinical, and inflammatory marker comparison by pathological group.

Parameter	Meningioma (*n* = 27)	Schwannoma (*n* = 12)	Ependymoma (*n* = 8)	Other (*n* = 17)	*p*
Age (years)	53.0 (37.0–68.0)	60.5 (49.5–68.8)	34.0 (25.8–40.8)	51.0 (44.0–71.0)	**0.023** ^a^
Sex, M/F	9/18	8/4	4/4	11/6	0.122 ^b^
Lesion level, C/T/L	6/18/3	1/2/9	1/0/7	0/4/13	**<0.001** ^b^
Motor deficit, Present	3 (11.1%)	4 (33.3%)	2 (25.0%)	7 (43.8%) ^c^	0.104 ^d^
NLR	2.77 (2.05–5.00)	1.94 (1.54–6.17)	2.21 (1.77–3.02)	2.61 (1.97–4.42)	0.506 ^a^
PLR	159.0 (114.9–219.0)	135.2 (116.7–172.6)	113.4 (97.0–145.8)	169.4 (122.7–222.6)	0.258 ^a^
LMR	3.80 (2.61–4.37)	3.39 (2.83–4.22)	4.30 (3.10–4.85)	3.57 (2.44–4.06)	0.573 ^a^
SII	760.0 (521.0–1601.5)	565.7 (441.8–1381.3)	518.7 (347.6–655.1)	782.7 (499.0–1350.0)	0.309 ^a^
PIV	415.9 (243.2–909.0)	408.5 (277.4–597.9)	269.6 (152.3–469.6)	424.0 (278.8–652.0)	0.568 ^a^
RDW (%)	41.5 (38.2–44.0)	42.2 (40.9–44.5)	40.1 (38.4–44.3)	40.0 (39.5–42.5)	0.621 ^a^

Continuous variables are presented as median (IQR: 25th–75th percentile); categorical variables as *n* (%). ^a^ Kruskal–Wallis test; Dunn post-hoc test with Bonferroni correction was applied for parameters reaching significance. ^b^ Chi-square test, confirmed with Fisher’s exact test because more than 25% of expected cell frequencies fell below 5. ^c^ The 1 patient with urinary incontinence (Other group) was excluded from motor deficit classification; motor deficit analysis in the Other group was performed on *n* = 16 (7/16 = 43.8%). ^d^ Chi-square test; results should be interpreted with caution as more than 25% of expected cell frequencies were <5. Bold *p*-values denote statistical significance at *p* < 0.05. M: male; F: female; C: cervical; T: thoracic; L: lumbar; NLR: neutrophil-to-lymphocyte ratio; PLR: platelet-to-lymphocyte ratio; LMR: lymphocyte-to-monocyte ratio; SII: systemic immune-inflammation index; PIV: pan-immune-inflammation value; RDW: red cell distribution width; IQR: interquartile range.

### 3.4. Sensitivity Analysis for Age Effect

To evaluate the potential confounding effect of age, Spearman correlation analyses were performed between age and each inflammatory marker within the patient cohort. No strong or clinically meaningful associations were identified for most inflammatory markers (NLR: r = 0.074, *p* = 0.562; PLR: r = 0.033, *p* = 0.798; LMR: r = 0.051, *p* = 0.688; SII: r = 0.010, *p* = 0.940; PIV: r = 0.046, *p* = 0.717). However, a moderate positive correlation was observed between age and RDW (r = 0.412, *p* = 0.001). These results suggest that the age imbalance between groups is unlikely to have substantially confounded the main findings, although residual confounding cannot be entirely excluded.

### 3.5. Relationship Between Motor Deficit and Inflammatory Markers

We found no difference in mean age between patients with and without motor deficit (55.8 ± 18.8 vs. 51.4 ± 17.3 years; *p* = 0.394). All six inflammatory markers—NLR, PLR, LMR, SII, PIV, and RDW—were similarly distributed in the two groups, with no marker approaching the significance threshold (all *p* > 0.05) (Table 4).

**Table 4:** Demographic characteristics and inflammatory markers by motor deficit status.

Parameter	Motor Deficit Present (*n* = 16)	Motor Deficit Absent (*n* = 47)	*p*
Age (years)	55.8 ± 18.8	51.4 ± 17.3	0.394 ^a^
NLR	2.41 (2.07–4.50)	2.53 (1.69–4.62)	0.522 ^b^
PLR	182.4 (114.0–227.3)	157.4 (111.8–187.6)	0.581 ^b^
LMR	3.79 (3.16–4.39)	3.62 (2.46–4.19)	0.425 ^b^
SII	767.5 (495.6–1523.0)	664.0 (429.0–1412.5)	0.507 ^b^
PIV	386.0 (247.1–609.6)	400.4 (247.4–652.5)	0.850 ^b^
RDW (%)	42.6 (39.7–46.1)	41.1 (39.2–43.8)	0.174 ^b^

Age is reported as mean ± SD; inflammatory markers as median (IQR: 25th–75th percentile). The patient with urinary incontinence was excluded from this analysis (*n* = 63). ^a^ Independent-samples *t*-test; age was normally distributed in both groups (Shapiro–Wilk: motor deficit present W = 0.904, *p* = 0.092; absent W = 0.959, *p* = 0.097). ^b^ Mann–Whitney U test. NLR: neutrophil-to-lymphocyte ratio; PLR: platelet-to-lymphocyte ratio; LMR: lymphocyte-to-monocyte ratio; SII: systemic immune-inflammation index; PIV: pan-immune-inflammation value; RDW: red cell distribution width; IQR: interquartile range; SD: standard deviation.

## 4. Discussion

Intradural spinal tumors are, by and large, benign—yet the compressive effects on the spinal cord and the potential for lasting neurological injury give them a clinical weight that their histology alone does not convey. We asked whether this patient population carries a detectable preoperative inflammatory signal in the peripheral blood, and whether that signal, if present, tracks with tumor histology or neurological status. The answer, at least in this series, is asymmetric: NLR, PLR, SII, and PIV were all elevated relative to controls, and LMR moved in the opposite direction, yet none of these differences mapped onto pathological subtype or motor deficit status. We interpret this pattern as evidence of a low-grade but consistent systemic immune shift that appears to accompany intradural spinal tumors regardless of their tissue of origin or clinical presentation.

The NLR elevation in our cohort—2.47 versus 2.06 in controls (*p* < 0.001)—deserves some contextual framing before drawing conclusions. In 153 patients with spinal metastases, NLR was reported at a median of 8.2 (IQR 4.0–14.0), with high values independently predicting both 30-day mortality (OR 5.20; *p* = 0.026) and shorter overall survival (HR 2.23; *p* = 0.003) [11]. Against that backdrop, the modest elevation we observed is entirely expected; the biology of metastatic spinal disease simply drives a far more intense systemic response. More directly comparable is the benign schwannoma literature, where an NLR cutoff of ≥2.21 emerged as an independent predictor of retreatment, while PLR in that same cohort failed to reach significance [12]. In a large meningioma series, NLR was marginally higher in the high-grade group on univariate analysis (2.41 vs. 2.16; *p* = 0.023) but lost independent predictive value in multivariate modeling, and PLR showed no association with tumor grade in any analysis [13]. A similar pattern appeared in brain metastasis patients, where NLR retained prognostic power that PLR could not replicate [14]. Across these data—and our own—NLR appears to capture something real about systemic immune activation even in the setting of benign intradural pathology, whereas PLR’s independent predictive value in central nervous system (CNS) tumors remains genuinely unsettled [15].

LMR was the only marker in our series that moved in the opposite direction from the rest, falling from a median of 4.34 in controls to 3.66 in patients (*p* < 0.001). The biological logic is not obscure: lymphocytes mediate antitumoral immune surveillance, while monocytes—as precursors of tumor-associated macrophages—tend to remodel the local microenvironment in ways that favor tumor persistence [16]. A low LMR may therefore reflect an early immunosuppressive shift rather than, or in addition to, an inflammatory one. Whether this shift carries prognostic weight is a separate question. In glioma, the evidence is not persuasive: a meta-analysis by Wang et al. returned a pooled hazard ratio of 0.82 (95% CI 0.60–1.12; *p* = 0.21), and LMR failed to associate with overall survival [17]. Li et al., however, found that LMR was significantly lower in malignant glioma (median 4.40) than in a benign meningioma control group (median 5.10), with an inverse correlation between LMR and tumor grade [18]. One might read that finding as evidence that low LMR only matters in the malignant context—but a meta-analysis restricted to benign meningiomas challenged that reading, demonstrating that higher-grade tumors within the benign spectrum still carried lower LMR than lower-grade counterparts (mean difference −0.82; *p* = 0.005) [19]. Given that roughly 80% of meningiomas in our series were WHO grade I, the fact that LMR remained depressed relative to healthy controls suggests that this suppression is a feature of intradural tumor biology more broadly, not a proxy for malignancy grade.

SII was elevated in our patients relative to controls (706.1 vs. 595.0; *p* = 0.003), and PIV separated the groups more sharply still (404.2 vs. 287.0; *p* < 0.001). The difference in statistical strength between the two indices is not surprising: PIV extends the SII formula by incorporating the monocyte count, and that additional cellular dimension appears to add discriminatory sensitivity. Across solid tumors broadly, high SII associates with poor overall survival—a 13-study meta-analysis reported a pooled hazard ratio of 1.80 (95% CI 1.43–2.28), with a median cutoff of 575 × 10^9^ [20]. In glioblastoma multiforme (GBM) specifically, an SII threshold above 510.8 × 10^9^ emerged as an independent adverse prognostic factor in multivariate analysis (HR = 1.672; *p* = 0.034) [21]. Yet a series of 79 high-grade glioma patients found no correlation between SII and either overall survival or time to recurrence, with the authors attributing the negative result to sample size and retrospective design [22]. That inconsistency alone argues against applying a universal SII cutoff to CNS tumors. What makes our data particularly difficult to reconcile with the existing literature is the comparison offered by Yang et al., who reported that SII and PIV did not differ from controls in benign intracranial tumors—meningiomas and acoustic neuromas—while both indices were clearly elevated in glioma [23]. Our series, predominantly benign intradural spinal tumors, produced significant SII and PIV elevations. We hypothesize that histopathological heterogeneity within our cohort and level-dependent local immune activation along the spinal axis may partly account for this divergence from the intracranial benign tumor data, though we cannot resolve this with the available evidence.

RDW did not differ between groups (41.8 ± 4.3 vs. 42.3 ± 3.5; *p* = 0.420). Unlike the other markers, RDW was normally distributed and analyzed by one-sample *t*-test. The null result is consistent with findings from GBM cohorts, where preoperative RDW similarly failed to predict overall survival in multivariate modeling; Kelly et al. attributed this to GBM’s intrinsic immunosuppressive environment blunting the pro-inflammatory pathways that RDW is thought to reflect, with the caveat that a significant postoperative rise in RDW was observed in that same cohort [24]. The malignant setting tells a different story: in surgery for brain metastases, an RDW cutoff of ≥13.2 independently predicted one-year mortality (HR 2.14; *p* < 0.001) [25]. The contrast between these series and our own reinforces the view that RDW’s behavior is tied to tumor burden and biological aggressiveness in ways that the predominantly benign intradural spinal tumor does not reach.

None of the six markers differentiated the pathological subgroups (all *p* > 0.05). Age and lesion level varied substantially across groups (*p* = 0.023 and *p* < 0.001, respectively), yet the immune profile remained flat. We interpret this uniformity as suggesting that the systemic inflammatory shift in intradural spinal tumors reflects a relatively non-specific host response rather than a signature of any particular tissue of origin. The heterogeneity of the “other” category—twelve distinct histopathological diagnoses—makes this interpretation even harder to push further. A large meningioma series similarly found that marker values clustered in overlapping ranges across high- and low-grade tumors, with PLR and several other parameters unable to predict grade independently [13]. The motor deficit comparison yielded the same pattern: age and all inflammatory markers were comparable between patients with and without deficit (all *p* > 0.05). Our data are consistent with the straightforward mechanistic argument that motor deficit in benign intradural tumors arises from mechanical cord compression, not from differential systemic immune activation—a comparison that, to our knowledge, has not been directly addressed in the prior literature.

The retrospective design and the relatively small sample constrain what can be concluded, particularly for the subgroup analyses; the ependymoma group, with only eight patients, was too small to support any subgroup-specific inference. Individual control data were unavailable, which forced the use of one-sample tests rather than standard two-sample methods for patient–control comparisons—a methodological compromise that should be kept in mind when reading the results. Age matching was imperfect: despite the intent to match on both age and sex, patient group age was significantly higher than control group age (*p* = 0.017), and this imbalance could influence the inflammatory marker levels to a degree that cannot be fully corrected post hoc. Against these constraints, the study does have features that strengthen its internal validity: all diagnoses were confirmed histopathologically, sex was perfectly matched between groups, and six inflammatory parameters were assessed simultaneously rather than selectively. Controlled comparisons of this kind—with a matched healthy reference group—remain uncommon in the intradural spinal tumor literature, and the findings provide a quantitative reference for future prospective work. Larger, multicenter studies with longer follow-up would allow these markers to be examined in relation to extent of resection, neurological recovery trajectory, and recurrence—dimensions that the present dataset cannot address. A key methodological concern is the age imbalance between groups. Additional analyses did not demonstrate a strong association between age and inflammatory markers, although residual confounding cannot be excluded.

Another limitation is the use of one-sample statistical tests due to the lack of individual-level control data, which may affect the robustness of statistical inference. Subgroup analyses are limited by relatively small sample sizes and should therefore be interpreted as inconclusive rather than definitive. The heterogeneity of the “other” group further limits the interpretation of subgroup comparisons. It is important to emphasize that these markers are not intended as diagnostic tools but may reflect systemic tumor-related inflammatory responses. Future studies integrating tumor-specific biomarkers, such as S-100, may provide further insight. Another limitation of this study is the lack of consistent long-term follow-up data due to its retrospective design.

## 5. Conclusions

Preoperative CBC-derived inflammatory markers are significantly altered in patients with intradural spinal tumors compared to healthy controls. Specifically, NLR, PLR, SII, and PIV were elevated, while LMR was decreased, supporting the presence of a consistent systemic inflammatory response associated with tumor presence.

Importantly, these markers did not demonstrate discriminatory capacity across pathological subtypes or neurological status, indicating that they likely reflect a non-specific host inflammatory response rather than tumor-specific biological characteristics. These findings are consistent with the observed heterogeneity of tumor histology and the absence of marker differentiation in subgroup analyses.

While these results provide insight into tumor-related systemic inflammation, the clinical applicability of these markers remains limited. They should therefore be interpreted as exploratory and hypothesis-generating. Future prospective studies incorporating larger cohorts, standardized statistical comparisons, integration of tumor-specific biomarkers, and long-term follow-up data are required to further clarify their potential role in clinical practice. Therefore, these markers should not be considered as standalone diagnostic tools but rather as supportive parameters within a broader clinical context.

## Figures and Tables

**Table 1:** Demographic and clinical characteristics of patients and controls.

Parameter	Patient Group (*n* = 64)	Control Group (*n* = 64)	*p*
Age (years)	51.5 (38.0–68.0)	47.0 ± 10.3	0.017 ^a^
Sex, M/F	32/32 (50%/50%)	32/32 (50%/50%)	1.000 ^b^
Lesion level			
Cervical	8 (12.5%)	—	—
Thoracic	24 (37.5%)	—	—
Lumbar	32 (50.0%)	—	—
Pathological diagnosis			
Meningioma	27 (42.2%)	—	—
Schwannoma	12 (18.8%)	—	—
Ependymoma	8 (12.5%)	—	—
Other ^c^	17 (26.6%)	—	—
Motor deficit, Present ^d^	16 (25.0%)	—	—
Motor deficit, Absent ^d^	47 (73.4%)	—	—
Urinary incontinence ^d^	1 (1.6%)	—	—
Complication, Present	1 (1.6%)	—	—
Recurrence, Present	4 (6.3%)	—	—

Continuous variables are presented as median (IQR: 25th–75th percentile) or mean ± SD; categorical variables as n (%). ^a^ One-sample Wilcoxon signed-rank test; age was not normally distributed (Shapiro–Wilk W = 0.959, *p* = 0.032). ^b^ Chi-square test; sex distribution was identical in both groups. ^c^ Other: cyst (*n* = 3), metastasis (*n* = 3), epidermal cyst (*n* = 2), mature cystic teratoma (*n* = 1), basosquamous cystic tumor (*n* = 1), teratoma (*n* = 1), angiolipoma (*n* = 1), paraganglioma (*n* = 1), lymphoma (*n* = 1), cavernous malformation/hemangioma (*n* = 1), lipoma (*n* = 1), chondrosarcoma (*n* = 1). ^d^ Motor deficit classification was performed in 63 patients; the 1 patient with urinary incontinence (Other group) was excluded from the Present/Absent motor deficit categories are explained in the table footnotes. M: male; F: female; IQR: interquartile range; SD: standard deviation.

**Table 2:** Comparison of systemic inflammatory markers between patient and control groups.

Marker	Patient Group (*n* = 64)	Control Group (*n* = 64)	*p*
NLR	2.47 (1.78–4.72)	2.06 (1.56–3.23)	<0.001 ^a^
PLR	157.1 (112.1–212.5)	121.0 (96.8–144.5)	<0.001 ^a^
LMR	3.66 (2.67–4.35)	4.34 (3.07–5.21)	<0.001 ^a^
SII	706.1 (445.8–1436.0)	595.0 (414.5–853.5)	0.003 ^a^
PIV	404.2 (249.6–634.0)	287.0 (210.5–507.5)	<0.001 ^a^
RDW (%)	41.8 ± 4.3	42.3 ± 3.5	0.420 ^b^

Continuous variables are presented as median (IQR: 25th–75th percentile) or mean ± SD. ^a^ One-sample Wilcoxon signed-rank test; control group medians were used as reference values because individual-level control data were unavailable. ^b^ One-sample *t*-test. NLR: neutrophil-to-lymphocyte ratio; PLR: platelet-to-lymphocyte ratio; LMR: lymphocyte-to-monocyte ratio; SII: systemic immune-inflammation index (neutrophil × platelet/lymphocyte); PIV: pan-immune-inflammation value (neutrophil × platelet × monocyte/lymphocyte); RDW: red cell distribution width; IQR: interquartile range; SD: standard deviation.

## Data Availability

The data that support the findings of this study are available from the corresponding author upon reasonable request. The data are not publicly available due to privacy or ethical restrictions.

## Abbreviations

The following abbreviations are used in this manuscript:

CBC	Complete Blood Count
CI	Confidence Interval
CNS	Central Nervous System
GBM	Glioblastoma Multiforme
HR	Hazard Ratio
IQR	Interquartile Range
LMR	Lymphocyte-to-Monocyte Ratio
MD	Mean Difference
NLR	Neutrophil-to-Lymphocyte Ratio
NSCLC	Non-Small Cell Lung Cancer
OR	Odds Ratio
PIV	Pan-Immune-Inflammation Value
PLR	Platelet-to-Lymphocyte Ratio
RDW	Red Cell Distribution Width
SD	Standard Deviation
SII	Systemic Immune-Inflammation Index
WHO	World Health Organization
